# miR-homoHSV of Singapore Grouper Iridovirus (SGIV) Inhibits Expression of the SGIV Pro-apoptotic Factor LITAF and Attenuates Cell Death

**DOI:** 10.1371/journal.pone.0083027

**Published:** 2013-12-03

**Authors:** Chuanyu Guo, Yang Yan, Huachun Cui, Xiaohong Huang, Qiwei Qin

**Affiliations:** 1 Key Laboratory of Tropical Marine Bio-resources and Ecology, South China Sea Institute of Oceanology, Chinese Academy of Sciences, Guangzhou, China; 2 University of Chinese Academy of Sciences, Beijing, China; 3 Department of Medicine, University of Alabama at Birmingham, Birmingham, Alabama, United States of America; Georgetown University, United States of America

## Abstract

Growing evidence demonstrates that various large DNA viruses could encode microRNAs (miRNAs) that regulate host and viral genes to achieve immune evasion. In this study, we report that miR-homoHSV, an miRNA encoded by Singapore grouper iridovirus (SGIV), can attenuate SGIV-induced cell death. Mechanistically, SGIV miR-homoHSV targets SGIV ORF136R, a viral gene that encodes the pro-apoptotic lipopolysaccharide-induced TNF-α (LITAF)-like factor. miR-homoHSV suppressed exogenous and endogenous SGIV LITAF expression, and thus inhibited SGIV LITAF-induced apoptosis. Meanwhile, miR-homoHSV expression was able to attenuate cell death induced by viral infection, presumably facilitating viral replication through the down-regulation of the pro-apoptotic gene SGIV LITAF. Together, our data suggest miR-homoHSV may serve as a feedback regulator of cell death during viral infection. The findings of this study provide a better understanding of SGIV replication and pathogenesis.

## Introduction

miRNAs are endogenous short RNAs, about 22 nt in length, which widely exist in plants, animals, and some viruses [1]. Similar to the biogenesis of mRNAs, miRNA precursors are mainly transcribed from genomic DNA by RNA polymerase II. Drosha and Dicer enzymes then successively process these precursors into mature miRNAs [2]. miRNAs negatively regulate target gene expression at the post-transcriptional level by either repressing translation or reducing the stability of target mRNA [3,4]. Increasing evidence indicates that miRNAs play pivotal roles in various cellular and physiological processes, such as development [5], metabolism [6], differentiation [7], cellular proliferation [8], and apoptosis [9–15]. 

Since the first discovery of viral miRNAs in Epstein-Barr virus (EBV) [16], there have been more than 250 viral miRNA sequences added to the miRbase database (version 19). Among these viral miRNAs, more than 90% come from DNA viruses, especially those in the *Herpesviridae* and *Polyomaviridae* families. These miRNA genes are expressed individually or in clusters, and a few share sequence homologies with each other or with host-encoded miRNAs. For example, seven rhesus lymphocryptovirus (rLCV) miRNAs are highly similar in sequence to EBV miRNAs encoded at a similar genomic location [17]. Previous studies suggested that viral miRNAs exert biological effects in two main aspects. First, viral miRNAs could inhibit host gene expression, thus affecting the host’s specific or nonspecific immune response. For example, human JC and BK polyoma viruses-encoded miRNAs could inhibit ULBP3 (NKG2D ligand) expression, thereby escaping NKG2D-dependent cell killing [18]. Second, some viral miRNAs could target virus-encoded genes to safeguard against inadvertent lytic reactivation. For example, EBV cluster 1-encoded miR-BART1-5p, as well as cluster 2-encoded miR-BART16 and miR-BART17-5p, could prevent apoptosis by targeting the EBV latent membrane protein 1 (LMP1) gene [19]. However, although a number of viral miRNAs have been identified, our knowledge of their function remains limited. Deeper investigations on viral miRNAs are needed to provide a better understanding of the mechanisms of viral infection and pathogenesis. 

SGIV, a novel member of the genus *Ranavirus* (family *Iridoviridae*), was first isolated from diseased brown-spotted grouper [20]. SGIV is a large double-stranded DNA virus with a spherical deoxyribonucleoprotein core surrounded by a lipid membrane [20]. The whole genome of SGIV has been sequenced and found to consist of 140,131 bp (GenBank accession number AY521625), with 162 predicted ORFs [21]. Our recent studies found that SGIV infection could induce typical apoptosis in non-host fathead minnow (FHM) cells, characterized by caspase activation, production of apoptotic bodies, and DNA fragmentation [22]. Furthermore, very recently, Yan et al. experimentally demonstrated that SGIV could encode miRNAs [23]. They identified 16 miRNAs from SGIV, including an miRNA (annotated as miR-homoHSV) located on the opposite strand within the SGIV ORF136R region (GenBank accession number YP_164231.1), sharing high sequence similarity with HSV-2 miR-H4-5p [23,24]. Previous work showed that the SGIV ORF136R encodes a novel lipopolysaccharide-induced TNF-α factor (LITAF) homolog, SGIV LITAF, which could induce apoptosis when over-expressed in FHM cells [25].

 During the process of co-evolution between host and virus, control over the cell death machinery is critical for survival of both host and virus [26]. To survive, viruses devised complicated regulatory mechanisms to modulate cell death mechanisms such as apoptosis. On one hand, many viruses promote apoptosis of host cells to spread virus progeny particles to neighboring cells and evade host inflammatory immune responses. On the other hand, to maintain latent and persistent infections, viruses block apoptosis to facilitate continued viral replication and maximize the production of progeny virus particles [27,28]. In the present study, we shed light on the molecular mechanisms of miR-homoHSV in the regulation of apoptosis and virus-induced cell death. We present data that miR-homoHSV could attenuate the effect of SGIV-induced cell death by targeting SGIV LITAF. Our findings highlight the important role of viral miRNAs in virus-induced cell death.

## Materials and Methods

### Cells and Virus

Grouper spleen (GS) cells [29] were grown in L15 medium containing 10% fetal bovine serum (FBS, Gibco, USA) at 25°C, and FHM cells [30] were grown in M199 medium containing 10% FBS at 25°C. SGIV (strain A3/12/98) was originally isolated from diseased brown-spotted grouper (*Epinephelus tauvina*), and the infection and propagation of SGIV in cell culture *in vitro* were performed as previously described [31].

### Vectors, RNA Oligoribonucleotides and Cell Transfection

 Our laboratory constructed all vectors used in this study. In brief, the full-length coding sequence of SGIV LITAF was amplified from SGIV genomic DNA and subcloned into the eukaryotic vector pEGFP-N3 to generate the SGIV LITAF expression vector pEGFP-LITAF [25]. The miR-homoHSV expression vector, pLL-homoHSV, was constructed by cloning an approximately 500 bp fragment of the miR-homoHSV precursor into *Bam*HI and *Xho*I sites of the pLL3.7 vector [23]. Four tandem repeats of a sequence perfectly complementary to miR-homoHSV was subcloned into the empty vector psiCHECK-2M located downstream of the *Renilla luciferase* gene to generate the dual-luciferase reporter vector, psiCHECK-homoHSV [23].

Custom miR-homoHSV mimics (mimic-homoHSV) and negative control (mimic-con), miR-homoHSV antagomir (antagomir-homoHSV) and negative control (antagomir-con) were purchased from RiboBio Company (RiboBio, China). The small interfering RNA (siRNA) targeting SGIV LITAF mRNA (si-LITAF, sense strand: 5′-CCAAAUGCAACAAUGUCAUCGCCGU-3′) and the control siRNA (si-con, sense strand: 5′-CAAGUAAAGACUGCCUAAUGAGAUA-3′) were purchased from Life Technologies (Invitrogen, USA).

Transfection of plasmid and miRNA mimics/antagomirs were performed using the Lipofectamine™ 2000 reagent and Lipofectamine™ RNAiMAX reagent, respectively, according to the manufacturer’s instructions (Invitrogen, USA). In brief, to detect the effect of miR-homoHSV on exogenous SGIV LITAF, 500 ng pLL-homoHSV/pLL3.7 and 300 ng pEGFP-LITAF were co-transfected into cells using Lipofectamine™ 2000 reagent. To detect the effect of mimic-homoHSV on exogenous SGIV LITAF, 800 ng pLL-homoHSV was transfected into cells using Lipofectamine™ 2000 reagent, followed by transfection of 20 pmol mimic-homoHSV/mimic-con using Lipofectamine™ RNAiMAX reagent. To detect the effect of antagomir-homoHSV on miR-homoHSV, 500 ng pLL-homoHSV and 300 ng pEGFP-LITAF were co-transfected into cells using Lipofectamine™ 2000 reagent, followed by transfection of 100 pmol antagomir-homoHSV/antagomir-con using Lipofectamine™ RNAiMAX reagent. To detect the effect of miR-homoHSV on endogenous SGIV LITAF, 800 ng pLL-homoHSV or 20 pmol mimic-homoHSV was transfected into cells before infected with SGIV. To detect the effect of si-LITAF on exogenous SGIV LITAF, 50 pmol si-LITAF/si-con was transfected into cells using Lipofectamine™ RNAiMAX reagent, followed by infection with SGIV.

### Selection of Stable Transfectants

 To obtain cells that stably express miR-homoHSV, GS cells were transfected with 800 ng pLL-homoHSV or empty vector pLL3.7 (as control) and then selected with 2 μg/ml puromycin (Sigma-Aldrich, USA). The transfection efficiency was monitored under a fluorescence microscope (Leica, Germany) by detection of the GFP expressed by the vector. After selective culture for 6 weeks, the percentage of GFP-expressing cells was nearly 90%. Then transfected cells were confirmed by stem-loop qRT-PCR to detect the expression of miR-homoHSV, as previously described [23]. The stable cell lines were named GS/pLL-homoHSV and GS/pLL-con. 

### Stem-loop Quantitative RT-RCR

 miRNAs were isolated from samples using the *mir*Vana™ miRNA Isolation Kit following the manufacturer's recommendations (Ambion, USA). Then the reverse transcription was carried out with special reverse transcriptase (RT) primers (designed by Invitrogen, USA). After the reverse-transcribed cDNA of each reaction was diluted five-fold with sterile water, real-time quantitative PCR based on the TaqMan MicroRNA assay was performed using the LightCyclerH 480 Detection System (Roche, Switzerland) and TaqManH microRNA Assay kit (Applied Biosystems, USA). All experimental data were normalized to the U6 gene. All experimental data were expressed as the means from at least three independent experiments. 

### Real-time PCR and Western Blot Analysis

 Total RNA was purified using the SV Total RNA Isolation System (Promega, USA) according to the manufacturer’s protocol. After DNase I digestion, cDNA was synthesized by the ReverTra Ace qPCR RT Kit (Toyobo, Japan). Then real-time PCR was performed using gene-specific primers. β-Actin was used as an internal control. All experiments were performed in triplicate. The primers used for real-time PCR are listed as follows, SGIV LITAF: 5′-GATGCTGCCGTGTGAACTG-3′ and 5′-GCACATCCTTGGTGGTGTTG-3′; SGIV MCP (GenBank accession number YP_164167.1): 5′-GCACGCTTCTCTCACCTTCA-3′ and 5′-AACGGCAACGGGAGCACTA-3′; SGIV ORF 016 (GenBank accession number YP_164111.1): 5′-GCCTTCCAGCGTTGATAT-3′ and 5′-GCGGTCCTATCTGTTCTC-3′; β-Actin (GenBank accession number HQ007251): 5′-TACGAGCTGCCTGACGGACA-3′ and 5′-GGCTGTGATCTCCTTCTGCA-3′.

Protein extracts were resolved by 10% SDS-PAGE and transferred to 0.45-μm PVDF membranes (Millipore, USA). Then the membranes were blocked with 5% skim milk for 1 h and washed three times in TBST. The mouse anti-SGIV LITAF or anti-SGIV MCP serum were used as primary antibodies and incubated with the membrane at a dilution of 1:2000 overnight at 4°C. After washing with TBST, the membrane was incubated with goat anti-mouse IgG coupled to alkaline phosphatase (1:10,000) (Sigma-Aldrich, USA) for 1 h at room temperature. All experiments were performed in triplicate. 

### Hoechst Staining

 To assess the effects of miR-homoHSV on SGIV-induced apoptosis, pLL-homoHSV or pLL3.7 was transfected into FHM cells 24 h before SGIV infection. Two days after SGIV infection, cell samples were washed with PBS and stained with Hoechst 33342 (Sigma-Aldrich, USA) at a final concentration of 1 μg/ml. The cells were observed under a fluorescence microscope to visualize nuclear morphology. 

The effects of miR-homoHSV on SGIV LITAF-induced apoptosis in FHM cells were also examined. pLL-homoHSV or pLL3.7 was co-transfected with pEGFP-LITAF into FHM. Forty-eight hours after transfection, Hoechst staining was performed and nuclear morphology was visualized as described above. 

### Detection of Sub-G0/G1 Cell Population with Flow Cytometry

 The proportion of apoptotic cells (cells in the sub-G0/G1 cell cycle fraction) was detected by flow cytometric analysis as previously described [32]. Briefly, cells were harvested and fixed in 70% pre-cooled ethanol overnight at −20°C. After washing with PBS and centrifugation at 500 × *g* for 10 min at room temperature, cells were stained with a 50-μg/ml propidium iodide (PI) solution (Sigma-Aldrich, USA) in the dark at room temperature for 30 min. Then PI fluorescence was measured by a FACScan flow cytometer (Becton-Dickinson, USA). Cells (1×10^4^) were analyzed for each sample. All experiments were performed in triplicate.

### Luciferase reporter gene assay

 Transient transfection was performed using Lipofectamine 2000 reagent or Lipofectamine™ RNAiMAX reagent (both from Invitrogen, USA), according to the manufacturer’s protocol. miR-homoHSV antagomir (100 pmol), 300 ng miR-homoHSV expression vector, and 100 ng psiCHECK-homoHSV were co-transfected into FHM cells. After 24 or 48 h, samples were harvested and luciferase activities detected with the Dual-Glo Luciferase Assay System (Promega, USA). All experiments were performed in triplicate.

### Viral Replication Kinetics Assay

 To determine the effect of miR-homoHSV on SGIV infection *in vitro*, viral replication kinetics was assessed based on SGIV multiplication in FHM cells transfected with pLL3.7 or pLL-homoHSV. Briefly, FHM cells were transfected with pLL3.7 or pLL-homoHSV for 24 h and then infected with SGIV at a multiplicity of infection (MOI) of 0.5. Cell lysates were collected at 24 and 48 h post infection (p.i.), serially diluted, and used to infect cells cultured in 96-well plates. Then the viral titers of the lysates were evaluated using the 50% tissue culture infectious dose (TCID(50)) assay [33]. Each sample was measured in triplicate. The appearance and changes in cytopathic effects (CPEs) were observed under a light microscope (Leica, Germany).

### Statistical Analysis

 All statistical results are presented as means ± standard deviations. The significance of differences between two groups was determined using Student’s *t*-test. A *P*-value <0.05 was deemed statistically significant.

## Results

### Effects of miR-homoHSV on Exogenous SGIV LITAF Expression

**Figure 1:**
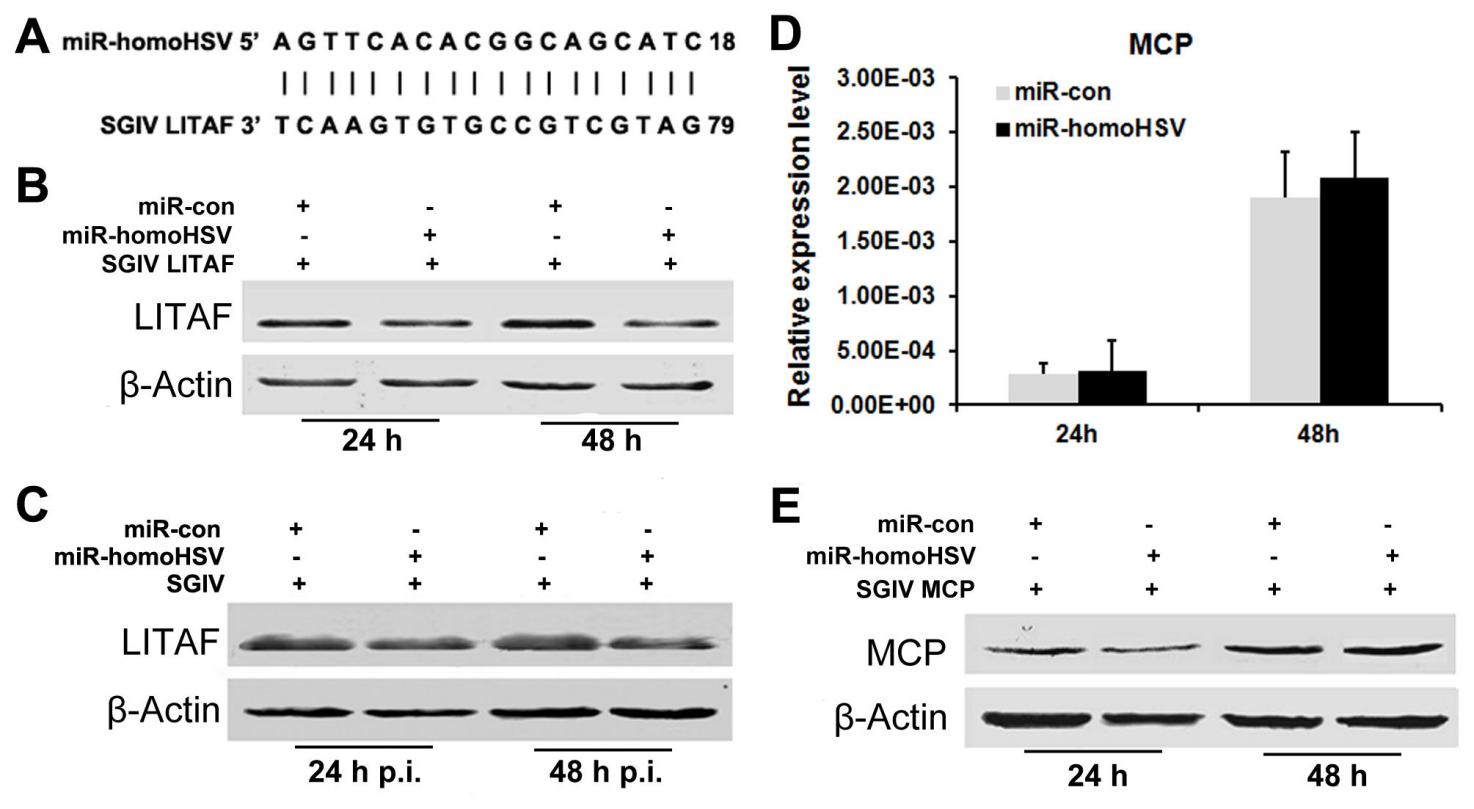
miR-homoHSV inhibits exogenous and endogenous SGIV LITAF expression. (A) miR-homoHSV located in the antisense strand of ORF136R. (B and C) miR-homoHSV inhibits exogenous (B) and endogenous (C) SGIV LITAF protein expression. (D and E) miR-homoHSV does not affect exogenous SGIV MCP expression at the mRNA (D) or protein (E) level.

Based on our previous study [23], we hypothesized that SGIV LITAF, which contains a sequence complementary to miR-homoHSV, is the putative target of miR-homoHSV (Figure 1A). To test this hypothesis, we studied the effect of miR-homoHSV on the expression of SGIV LITAF from an overexpression vector. 

After the transfection efficiency and expression of miR-homoHSV was confirmed (Figure S1), the effect of miR-homoHSV was examined. SGIV LITAF expression was inhibited by miR-homoHSV, as observed at the protein level (Figure 1B). In addition, a GS cell line was created that stably expresses miR-homoHSV and a stable cell line transfected with the empty vector served as a control. Two cell lines were transfected with the SGIV LITAF expression vector and compared for their levels of SGIV LITAF. Concordant with the results observed in FHM cells, miR-homoHSV expression inhibited the SGIV LITAF protein expression (data not shown).

To rule out the possibility that the expression difference observed is due to inconsistent transfection efficiency, SGIV ORF072R (encoding SGIV major capsid protein, MCP) was selected as a negative control. Real-time PCR and western blot results revealed that neither mRNA levels nor the protein levels of SGIV ORF072R (MCP) were affected by miR-homoHSV expression (Figure 1D and E). These results suggest that miR-homoHSV specifically targets SGIV LITAF.

**Figure 2:**
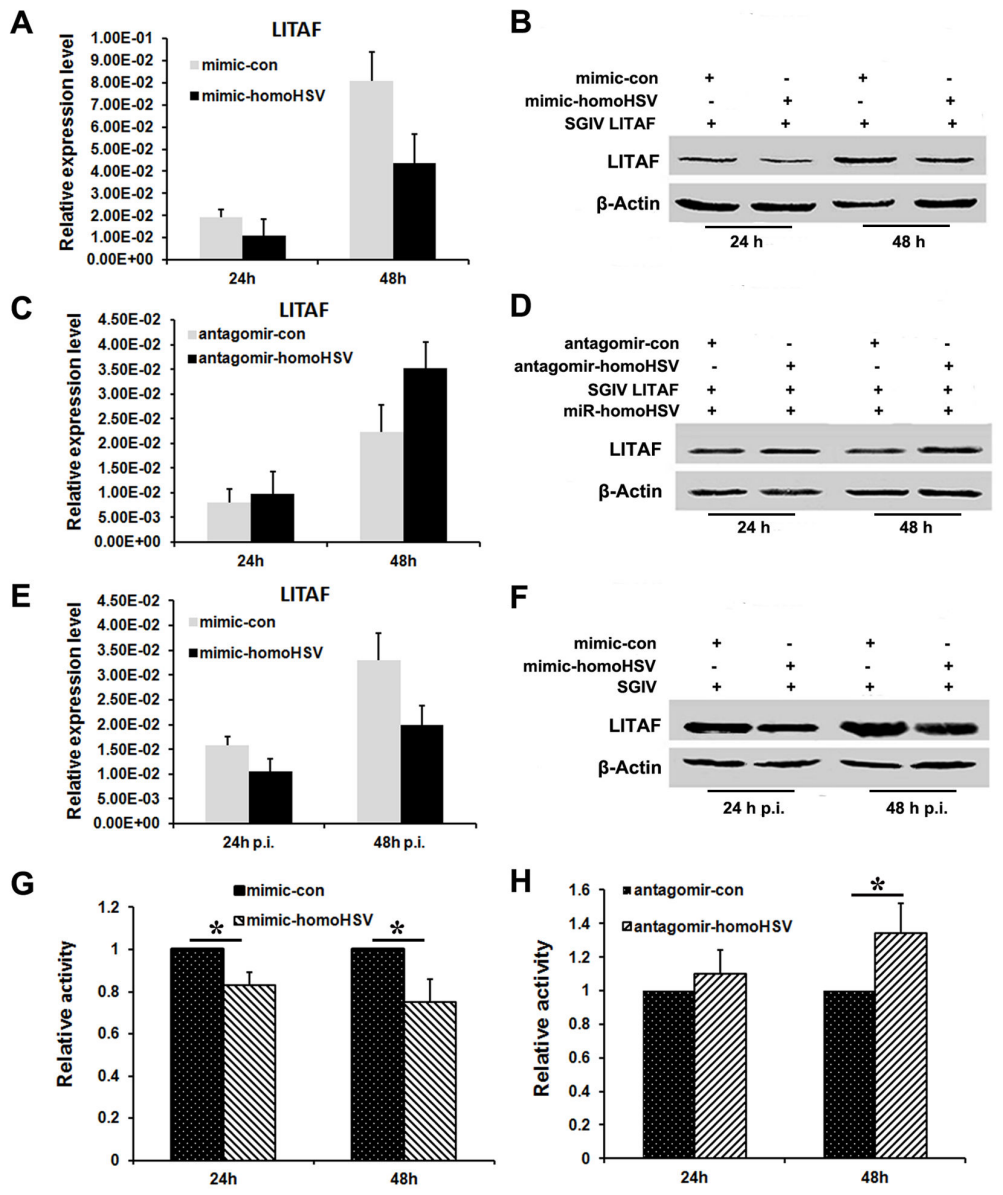
Effects of mimic-homoHSV and antagomir-homoHSV on SGIV LITAF. (A and B) Suppression of exogenous SGIV LITAF mRNA (A) and protein (B) expression by mimic-homoHSV. (C and D) Antagomir-homoHSV attenuates the effects of miR-homoHSV on exogenous SGIV LITAF mRNA (C) and protein (D) expression. (E and F) Suppression of endogenous SGIV LITAF mRNA (E) and protein (F) expression by mimic-homoHSV. (G and H) Analysis of luciferase activity. mimic-homoHSV and psiCHECK-homoHSV were co-transfected into FHM cells (G); antagomir-homoHSV, miR-homoHSV expression vector, and psiCHECK-homoHSV were co-transfected into FHM cells (H). Data are means from at least three independent experiments done in duplicate. Error bars indicated the standard error of the mean (SEM). *P<0.05, compared with cells co-transfected with mimic-con or antagomir-con.

Moreover, miR-homoHSV mimics (mimic-homoHSV) and miR-homoHSV antagomirs (antagomir-homoHSV) were employed to further study the effect of miR-homoHSV on SGIV LITAF in FHM cells. Concordantly, miR-homoHSV mimics were able to reduce the levels of SGIV LITAF expressed by the overexpression vector (Figure 2A and B), while miR-homoHSV antagomirs attenuated the inhibition of miR-homoHSV to SGIV LITAF (Figure 2C and D). 

A previous study showed that vector-based expression of miR-homoHSV could markedly reduce the luciferase activities from a psiCHECK-homoHSV reporter [23]. The effects of mimic-homoHSV and antagomir-homoHSV on the psiCHECK-homoHSV reporter were evaluated. Results revealed that compared with the control groups, mimic-homoHSV decreased the relative luciferase activities of psiCHECK-homoHSV (Figure 2G), while antagomir-homoHSV increased the reporter activities, demonstrating its attenuation of inhibition by miR-homoHSV on the psiCHECK-homoHSV reporter (Figure 2H). These data provide further evidence that miR-homoHSV suppressed SGIV LITAF expression by targeting its complementary sequence in the SGIV LITAF gene.

### Effects of miR-homoHSV on Endogenous SGIV LITAF Levels

The ability of miR-homoHSV to reduce endogenous SGIV LITAF expression in the context of virus infection was tested. FHM cells were transfected with miR-homoHSV expression vector 24 h before infection with SGIV and total proteins were collected at 24 and 48 h p.i. Western blot results revealed that endogenous SGIV LITAF protein levels were down-regulated by miR-homoHSV expression (Figure 1C). This was consistent with results observed in co-transfection experiments. In addition, the effects of miR-homoHSV mimics on the endogenous expression of SGIV LITAF were examined. Results showed that miR-homoHSV mimics repressed endogenous SGIV LITAF expression at both mRNA and protein levels (Figure 2E and F).

### Roles of miR-homoHSV in SGIV LITAF-Induced Apoptosis

**Figure 3:**
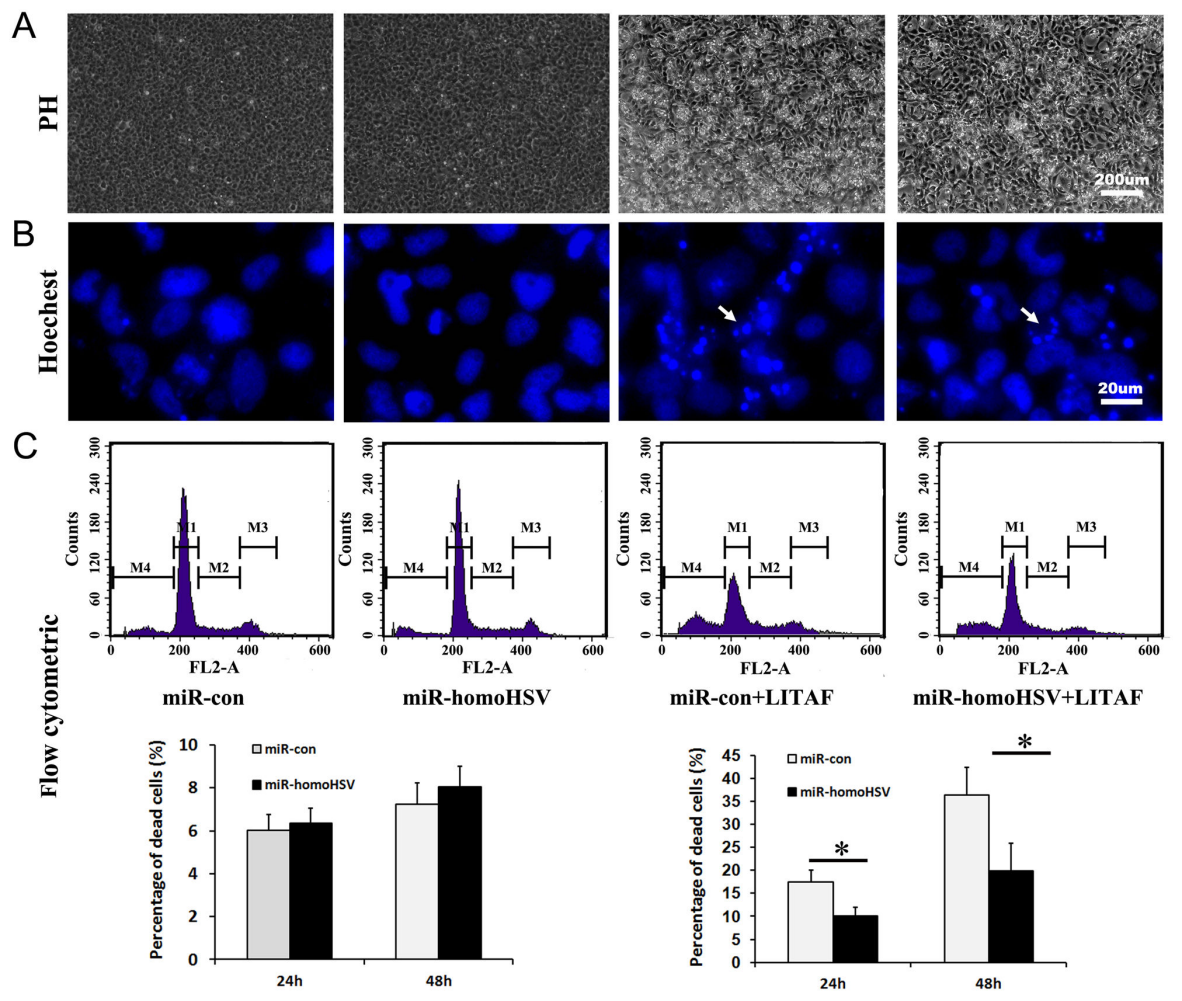
miR-homoHSV inhibits SGIV LITAF-induced apoptosis. (A) Morphology of FHM cells 48 h after transfection with the empty vector pLL3.7, pLL-homoHSV, pLL3.7 and pEGFP-LITAF, and pLL-homoHSV and pEGFP-LITAF, from left to right. (B) Nuclear morphology of FHM cells 48 h after transfection with the same vectors above. Arrows indicate apoptotic bodies. Many apoptotic bodies were observed in cells co-transfected with pLL3.7 and pEGFP-LITAF. (C) Flow cytometric analysis of dead cells in FHM cells transfected with the vectors above 48 h after transfection. (D) Statistical results for the proportion of PI-stained positive cells 24 h and 48 h after transfection with empty vector pLL3.7, pLL-homoHSV (left panel), pLL3.7 and pEGFP-LITAF, and pLL-homoHSV and pEGFP-LITAF (right panel). Data are means from at least three independent experiments. Error bars indicate SEM. *P<0.05.

As SGIV LITAF overexpression could induce cell apoptosis in FHM cells [25], miR-homoHSV was examined for its ability to regulate SGIV LITAF-induced cell apoptosis. FHM cells were co-transfected with the miR-homoHSV expression vector (pLL-homoHSV) and the SGIV LITAF expression vector (pEGFP-LITAF). As a control, cells were transfected with pEGFP-LITAF and pLL3.7 empty vector. At 24 h and 48 h after transfection, cellular and nuclear morphologies were observed under a microscope before and after staining by Hoechst 33342 (Figure 3A and B). Compared with the control groups, the degree of apoptosis in cells co-transfected with pLL-homoHSV and pEGFP-LITAF was much lower, demonstrated by the reduced numbers of dead cells and apoptotic bodies, while there was no difference between cells transfected with pLL-homoHSV and pLL3.7 alone. Flow cytometric analysis also confirmed this observation (Figure 3C and D). 

### Functions of miR-homoHSV in Viral Biology

**Figure 4:**
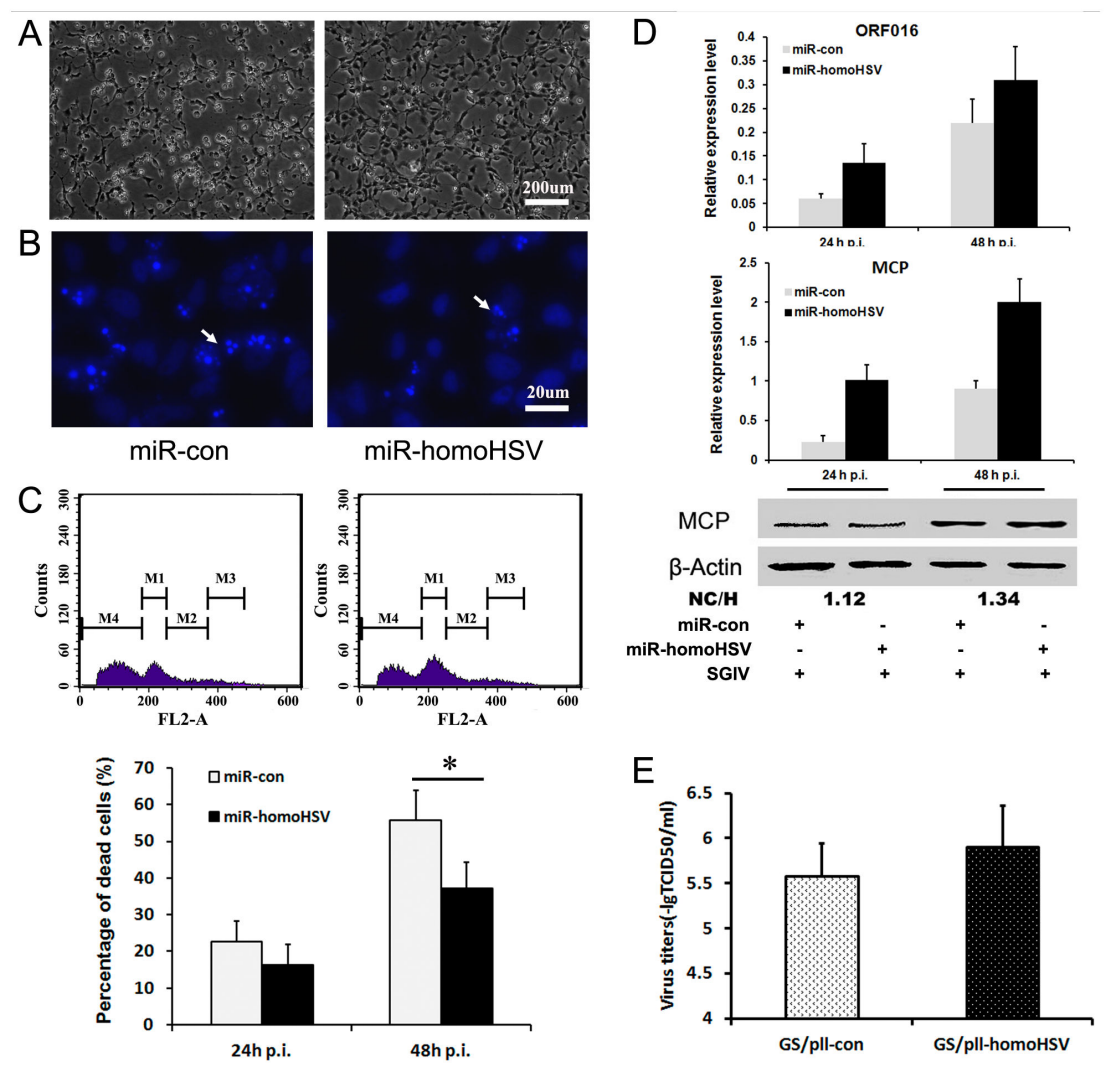
miR-homoHSV attenuates SGIV-induced apoptosis and promotes SGIV replication. (A) Morphology of FHM cells 48 h after infection with SGIV. FHM cells was transfected with pLL3.7 (left) or pLL-homoHSV (right), and then infected with SGIV. (B) Nuclear morphology of FHM cells 48 h after infection with SGIV. Cells were treated as described for panel A. Arrows indicate apoptotic bodies. Many apoptotic bodies were observed in pLL3.7 transfected FHM cells (left). (C) Flow cytometric analysis of dead cells in FHM cells treated as described for panel A. Top panels are PI staining and fluorescence-activated cell sorter analysis of FHM cells 48 h after infection. Bottom panel shows the statistical results of the proportion of PI-stained positive cells 24 h and 48 h after SGIV infection. Data are means of at least three independent experiments; error bars indicate SEM. *P<0.05. (D and E) miR-homoHSV promotes endogenous SGIV ORF016L (D) and SGIV MCP (E) mRNAs (top) and SGIV MCP protein (bottom) expression in FHM cells. (F) miR-homoHSV promotes SGIV replication. FHM cells overexpressing miR-homoHSV/miR-con were infected with SGIV, and virus was collected 48 h p.i. The viral titer was measured using the TCID50 method. Error bars indicate SEM.

To explore the roles of miR-homoHSV during SGIV infection, experiments were performed from both cellular and viral aspects. For the impacts of miR-homoHSV on cellular progress, FHM cells were transfected with the miR-homoHSV expression vector or control vector, followed by SGIV inoculation at 24 h after transfection. Hoechst staining was then performed and the CPE and apoptotic bodies compared at 24 and 48 h p.i. Cells transfected with miR-homoHSV expression vector exhibited less severe CPE and relatively fewer apoptotic bodies compared to the control (Figure 4A and B). Furthermore, flow cytometric analysis showed that apoptotic cell population at sub-G0/G1 was lower in miR-homoHSV expression cells, especially at 48 h p.i. (Figure 4C). 

Furthermore, the effect of miR-homoHSV expression on viral replication was detected. Two viral genes (ORF016L and ORF072R) encoded viral structure protein were selected. Transcript levels of SGIV ORF016L and ORF072R (MCP), as well as ORF072R (MCP) protein levels were higher in miR-homoHSV expression cells than the control group after viral infection (Figure 4D and E). Viral titer assays further showed that miR-homoHSV expression cells produced much more infectious SGIV particles (Figure 4F). These data suggest that the expression of miR-homoHSV plays a beneficial role during viral infection.

### miR-homoHSV Blocks SGIV-induced Cell Death via Targeting SGIV LITAF

**Figure 5:**
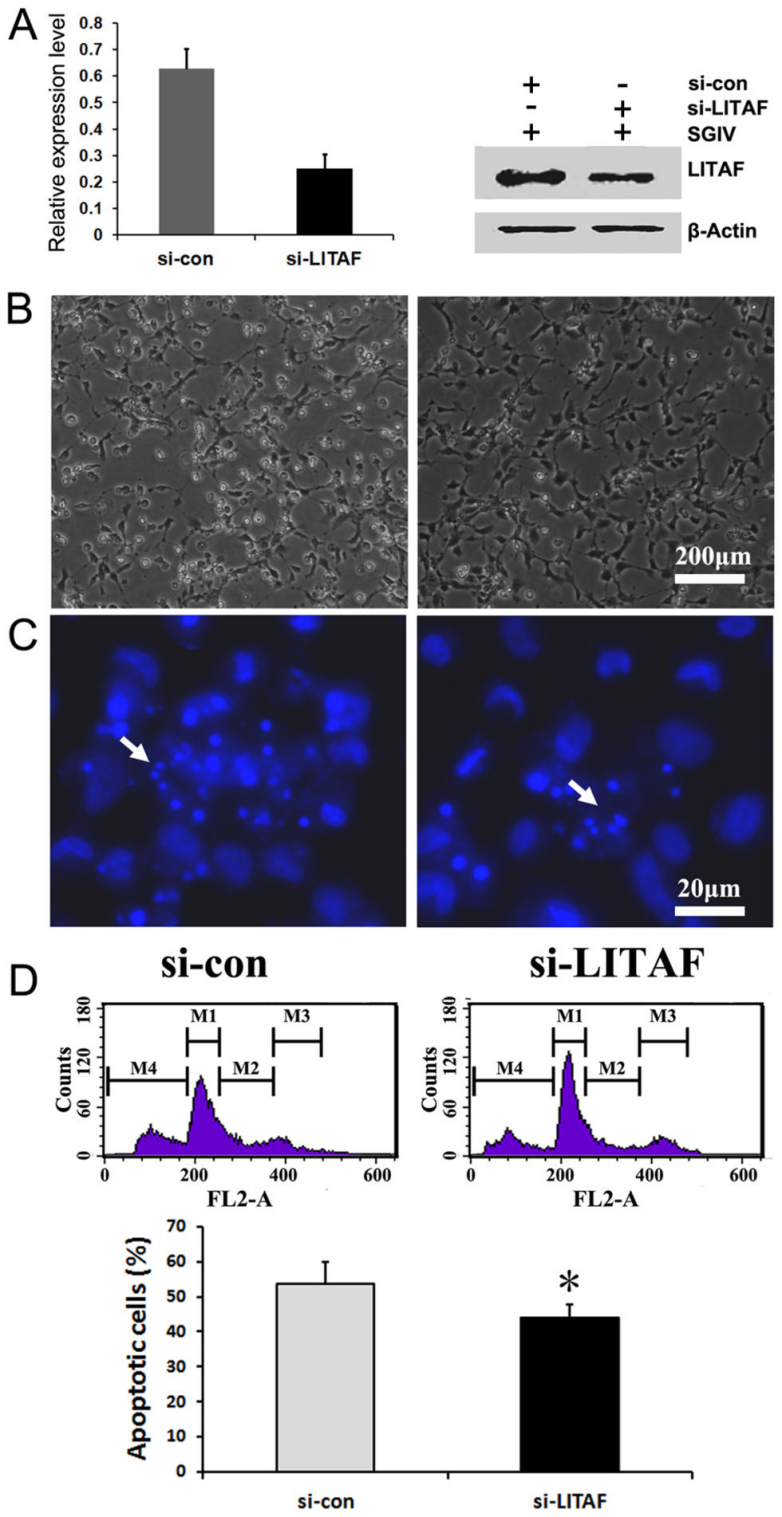
si-LITAF specifically blocks SGIV-induced apoptosis. (A) si-LITAF efficiently suppresses endogenous SGIV LITAF mRNA (left) and protein (right) expression in FHM cells. (B and C) Morphology (B) and nuclear morphology (C) of FHM cells 48 h after infection with SGIV. FHM cells were transfected with si-con (left) or si-LITAF (right), and infected with SGIV 18 h later. Arrows (bottom panels) indicate apoptotic bodies. Many apoptotic bodies were observed in si-con transfected FHM cells. (D) si-LITAF blocks SGIV-induced apoptosis. PI staining and fluorescence-activated cell sorter analysis of FHM cells 48 h after infection (up). Statistical results of the proportion of PI-stained positive cells 48 h after infection with SGIV (bottom). Data are means of at least three independent experiments; error bars indicate SEM. *P<0.05. Flow cytometric analysis of apoptotic cells in FHM cells treated as described for panel B.

SGIV LITAF was previously demonstrated as an authentic target of miR-homoHSV. Therefore the blockade of SGIV-induced cell death by miR-homoHSV may be dependent on its direct targeting of SGIV LITAF. To answer this, a small interfering RNA (siRNA)-based knockdown strategy was used to block the expression of SGIV LITAF. FHM cells were transfected with si-LITAF and infected with SGIV. Endogenous SGIV LITAF mRNA and protein expression were analyzed at 24 and 48 h p.i. The siRNA against SGIV LITAF was able to efficiently inhibit the expression of SGIV-LITAF by >50% (Figure 5A). Furthermore, the inhibition of SGIV LITAF blocked SGIV induced cell death, as indicated by the results of microscope observation, Hoechst staining assay, as well as flow cytometric analysis (Figure 5B–D). Together, these data indicate that SGIV miR-homoHSV may at least partially inhibit viral-induced cell death through targeting the SGIV LITAF gene.

### Expression Profiles of Endogenous miR-homoHSV and SGIV LITAF

**Figure 6:**
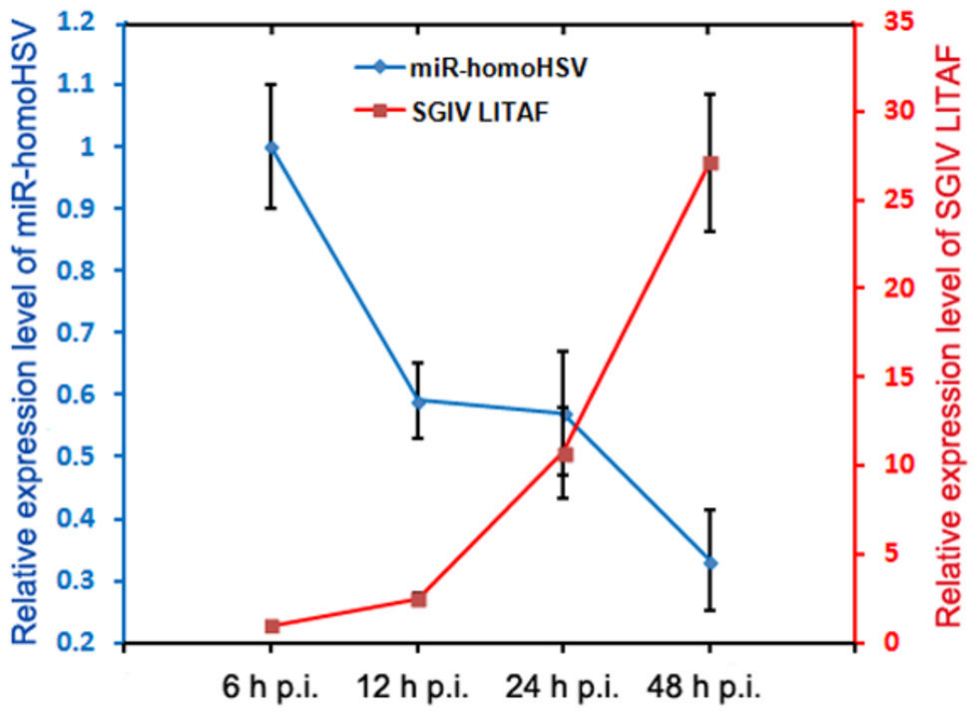
Expression profiles of endogenous miR-homoHSV and SGIV LITAF. The relative expression level of miR-homoHSV rose rapidly reaching its peak 6 h p.i. and then decreased (blue). Relative expression level of miR-homoHSV 6 h p.i. was set to 1 for comparison. The U6 gene was used as an internal control. The amount of SGIV LITAF mRNA accumulated stably from 6 to 48 h p.i. (red). Relative expression level of SGIV LITAF 6 h p.i. was set to 1 for comparison. β-Actin was used as an internal control. Data are means from rapidly at least three independent experiments done in duplicate; error bars indicate SEM.

 To get an overview of endogenous miR-homoHSV and SGIV LITAF expression profiles during viral infection, virus-infected GS cells were harvested at different time points after infection. The miRNAs and mRNAs were extracted from these samples, and real-time PCR assays were performed. Results revealed a negative correlation between the expression of miR-homoHSV and SGIV LITAF. The relative expression level of miR-homoHSV rose rapidly reaching its peak 6 h p.i., and then decreased by about 70% over the subsequent 36 hours. While the amount of SGIV LITAF mRNA accumulated stably from 6 to 48 h p.i. (Figure 6). SGIV LITAF expression coincided with a reduction of the expression of miR-homoHSV, indicating that SGIV may encode miR-homoHSV to inhibit the levels of SGIV LITAF mRNA at an early stage of infection.

## Discussion

As obligatory intracellular pathogens, viruses possess the ability to utilize the biosynthetic machinery of host cells and evade host defenses, such as humoral responses, interferons, cytokines and chemokines, and apoptotic responses [34–39]. Previous studies extensively focused on identifying viral-encoded proteins that specifically inactivate host cell defense responses. Recently, increasing evidence has shown that many viruses encode miRNAs to regulate the expression of specific genes to achieve immune escape. For example, human cytomegalovirus (HCMV) miR-UL112 inhibits the gene expression of major histocompatibility complex class I-related chain B (MICB), leading to the reduced killing of virus-infected cells by NK cells [40]. Kaposi’s sarcoma-associated herpesvirus (KSHV) encodes miR-K12-1, promoting viral latency by targeting the IκBα host survival pathway gene [41]. However, there are still a large number of viral miRNAs awaiting experimental characterization. In this study, the role of SGIV-encoded miR-homoHSV in the regulation of virus-induced cell death was examined. To our knowledge, this is the first report describing an miRNA encoded by a marine vertebrate virus that could target a viral transcript to attenuate virus-induced cell death.

To date, four predominant strategies (antisense targets, computational prediction, RISC pull-down, and phenotype screens) have been used to uncover the function of viral miRNAs [42]. Since miR-homoHSV is located in the antisense strand of ORF136R, which encodes SGIV LITAF, we hypothesized that SGIV LITAF is a potential target of miR-homoHSV. To confirm this hypothesis, the effects of miR-homoHSV on SGIV LITAF protein expression levels were examined through several cell-based experiments. We found that the overexpression of miR-homoHSV could inhibit both endogenous and exogenous protein expression derived from the SGIV LITAF gene, while the specific inhibition of miR-homoHSV by antagomirs restored SGIV LITAF expression at both mRNA and protein levels. 

Due to its full complementarity to a target gene, miRNAs have been predicted to suppress target RNAs in an siRNA-like cleavage manner [43–46]. For example, HSV2 miR-I is fully complementary to the neurovirulence factor gene ICP34.5 and can cleave ICP34.5 mRNA in an siRNA-like manner [47]. We found that synthesized miR-homoHSV mimics could decrease SGIV LITAF expression at both mRNA and protein levels. Thus, we hypothesized that SGIV miR-homoHSV may regulate SGIV LITAF expression through direct cleavage of SGIV LITAF mRNA. Further experiments will determine whether the knockdown of Drosha has a similar phenotype as the miR-homoHSV antagomir. 

Accumulating evidence has revealed that miRNAs could regulate apoptosis through targeting pro- or anti-apoptotic genes, such as MOAP1, caspases, and death receptors [48–53]. Our recent work found that SGIV LITAF overexpression induces cellular apoptosis, indicating SGIV LITAF is a pro-apoptotic factor [25]. As miR-homoHSV targets SGIV LITAF, the ability of miR-homoHSV to regulate SGIV LITAF-induced apoptosis was examined. After co-transfection of the miR-homoHSV expression vector and pEGFP-LITAF into FHM cells, SGIV LITAF-induced apoptosis was inhibited, as demonstrated by decreases in apoptotic bodies and the proportion of dead cells. The paradox that an SGIV-encoded miRNA inhibits viral gene function suggests a proactive role for SGIV in the regulation of cellular processes. Considering that miRNA act as rheostats to modulate the output of gene expression [54], one reasonable explanation for the paradox is that miR-homoHSV may inhibit aberrantly transcribed SGIV LITAF mRNA in the process of viral infection to facilitate a complete viral life cycle.

Furthermore, to examine the role of miR-homoHSV in viral infection, miR-homoHSV expression was enforced in FHM cells and followed by SGIV infection. Intriguingly, compared with the control group, SGIV-induced cell death was significantly attenuated by miR-homoHSV expression. Meanwhile, the expression of viral structural proteins was enhanced. In consideration of the suppressive effects of miR-homoHSV on SGIV LITAF expression and SGIV LITAF-induced apoptosis, we hypothesized that through targeting SGIV LITAF, miR-homoHSV regulates virus-induced cell death, thus facilitating viral replication. An siRNA was employed to further determine the role of SGIV LITAF during SGIV infection. Real-time PCR and western blot results showed that the expression of SGIV LITAF was efficiently blocked by siRNA. Furthermore, SGIV-induced apoptosis was attenuated by si-LITAF oligos. Consequently, the anti-apoptosis effect of miR-homoHSV relies, at least partially, on its ability to down-regulate SGIV LITAF expression. 

Similar phenomena have been reported previously. EBV miR-BART2, located directly antisense to the viral DNA polymerase BALF5, was found to be required for viral replication during the lytic cycle and absent in the latent cycle. The regulation of miR-BART2 on BALF5 is thus dependent on the viral life cycle. In the lytic viral replication cycle, the miR-BART2 level was down-regulated, leading to the reduced inhibition of BALF5 mRNA expression, while forced expression of miR-BART2 during lytic replication caused a reduction in BALF5 protein levels [55]. In another example, ICP4 is an immediate early gene important for infectious laryngotracheitis virus (ILTV) growth. Through down-regulation of ICP4 by siRNA-silencing, iltv-miR-I5, mapped antisense to the ICP4 gene, was found to affect the balance between lytic and latent states of the virus *in vivo* [43]. 

For our study, although it remains unclear whether SGIV has a latent state during infection *in vivo*, there is a possibility that control of SGIV LITAF expression in virus-infected cells by miR-homoHSV affects cell processes and the final outcome of mature viruses. As indicated by the expression profiles of endogenous miR-homoHSV and SGIV LITAF during viral infection, we concluded that at the early stage of infection, miR-homoHSV may inhibit SGIV LITAF expression and cellular apoptosis, facilitating the replication of viral particles assembly. This inhibitory effect, weakened at a late stage, results in the release of virus. The similar regulation mechanism was reported in *Heliothis virescens* ascovirus (HvAV) [56]. There was a coincidence between HvAV-miR-1 expression and a marked reduced expression of target, HvAV DNA polymerase I.

The degree of inhibition by miR-homoHSV on virus-induced cell death was lower than that from SGIV LITAF induction, indicating the existence of other pro-apoptotic factors not regulated by miR-homoHSV in the progress of viral induced apoptosis. Moreover, these data indicate SGIV genes, including both protein-coding genes and noncoding RNAs (ncRNAs), may work synergistically in a highly complicated manner to benefit viral pathogenesis, such as through evading immune surveillance, and promoting viral replication and release. 

In conclusion, the viral miRNA, SGIV miR-homoHSV, attenuates viral-triggered cell death by regulating the pro-apoptotic viral gene SGIV LITAF *in cis*. SGIV miR-homoHSV functions in a negative feedback loop to prevent excessive cell death induced by viral infection, which is beneficial for viral replication and assembly in the infected cells. Our data provide useful clues for understanding SGIV pathogenesis, and may help in the development of novel strategies for combating SGIV infection.

## Supporting Information

Figure S1 **miR-homoHSV is expressed in FHM cells.** (A and B) The transfection efficiency for pLL3.7 (A) and pLL-homoHSV (B) was monitored through the detection of GFP expression. (C) The expression of miR-homoHSV was detected by stem-loop qRT-PCR. The U6 gene was used as an internal control.
(TIF)
