# Supplementary figures and images for: miR-homoHSV of Singapore Grouper Iridovirus (SGIV) Inhibits Expression of the SGIV Pro-apoptotic Factor LITAF and Attenuates Cell Death
Source: PLoS One. 2013 Dec 3;8(12):e83027. doi: 10.1371/journal.pone.0083027 (PMC3849457; doi:10.1371/journal.pone.0083027)

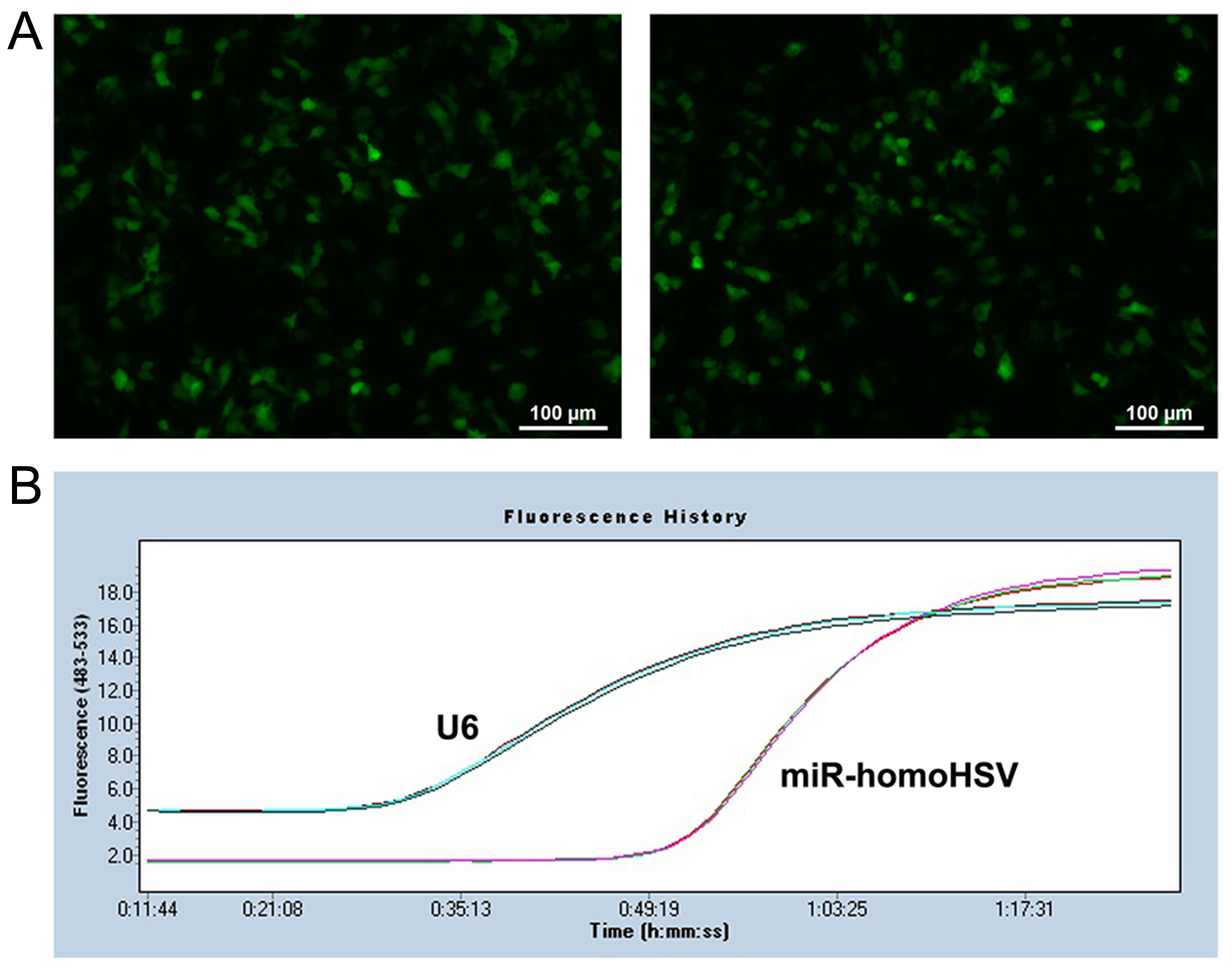

Supplement: Figure S1 — miR-homoHSV is expressed in FHM cells. (A and B) The transfection efficiency for pLL3.7 (A) and pLL-homoHSV (B) was monitored through the detection of GFP expression. (C) The expression of miR-homoHSV was detected by stem-loop qRT-PCR. The U6 gene was used as an internal control. (TIF) [file pone.0083027.s001.tif]
